
JOURNAL INFORMATION
==============================
NLM Title Abbreviation: ScientificWorldJournal
Journal ID: ScientificWorldJournal
NLM Journal ID: 101131163
Journal ID: TSWJ

The Scientific World Journal

ISSN: 2356-6140
EISSN: 1537-744X
Publisher: Wiley

ARTICLE INFORMATION
==============================
PMCID: PMC5763898
PMID: 20842315
DOI: 10.1100/tsw.2010.175
Article ID: 748384
Article version: 1
Subjects: Clinical Images

Dermoscopy of Tick Bite

Rallis Efstathios *
Department of Dermatology Veterans Administration Hospital (NIMTS), Athens, Greece
*Efstathios Rallis: efrall@otenet.grAcademic Editor: Daniel Batlle

Publication date: 2010
Electronic publication date: 2010 Sep 1
Volume: 10
First page: 1705
Last page: 1706
Received 2010 Jul 29; Revised 2010 Aug 12; Accepted 2010 Aug 16
Copyright: Copyright © 2010 Efstathios Rallis.
Copyright year: 2010
License: This is an open access article distributed under the Creative Commons Attribution License, which permits unrestricted use, distribution, and reproduction in any medium, provided the original work is properly cited.
License URL: https://creativecommons.org/licenses/by/3.0/

Keywords: dermoscopy, tick

==============================
Clinical Image
*TheScientificWorldJOURNAL* (2010) 10, 1705–1706
ISSN 1537-744X; DOI 10.1100/tsw.2010.175

TheScientificWorldJOURNAL
www.thescientificworld.com

### Dermoscopy of Tick Bite

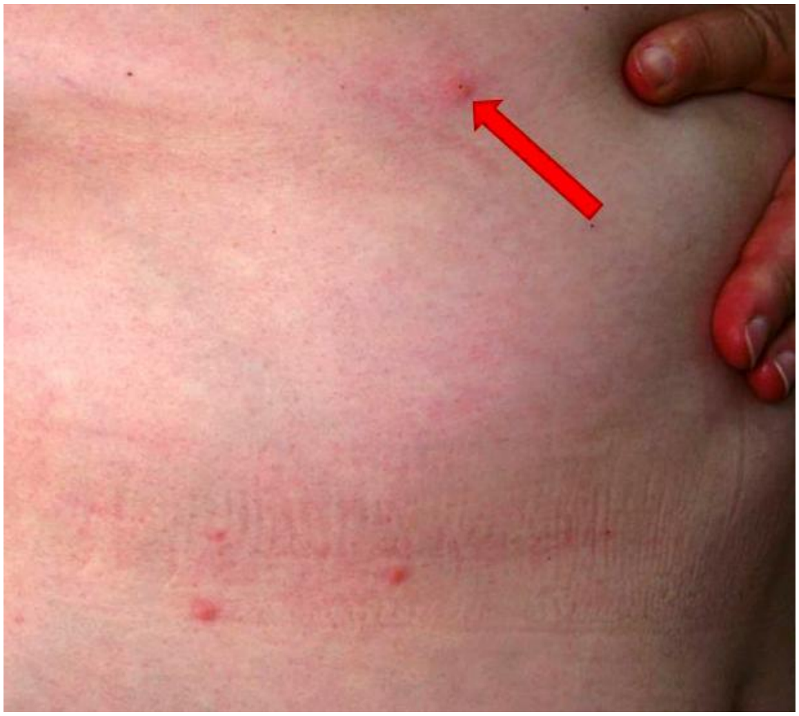

**FIGURE 1.** Numerous, intensively pruritic, erythematous papules on the patients' trunk. The arrow shows the site of the dermoscopic picture.

Efstathios Rallis

*Department of Dermatology, Veterans Administration Hospital (NIMTS), Athens, Greece*

**E-mail**: efrall@otenet.gr

*Received July 29, 2010; Revised August 12, 2010; Accepted August 16, 2010; Published September 1, 2010*

**KEYWORDS**: dermoscopy, tick

A 54-year-old woman presented with a 12-h history of the appearance of numerous, intensively pruritic, erythematous papules on her trunk (Fig. 1). Dermoscopy showed the presence of small-sized ticks in the majority of lesions (Fig. 2). By using medium-tipped, angled forceps, every tick was grasped as close to the skin as possible and tracked out vertically. A total of 28 ticks were finally removed.

©2010 with author.

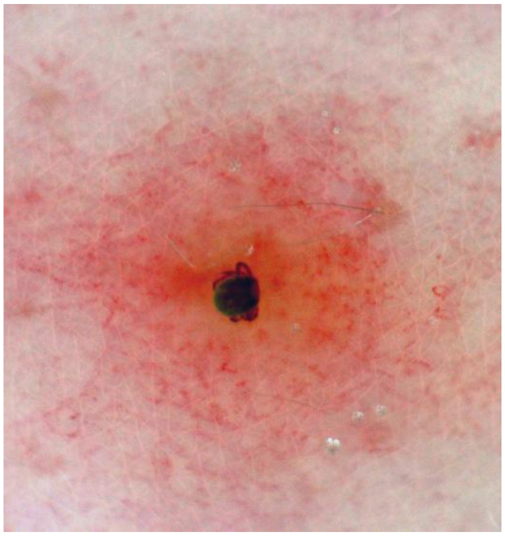

**FIGURE 2.** Dermoscopic findings of tick bite.

Ticks are blood-feeding ectoparasites usually found in grass and woods, and on pets. They harbor and spread a variety of pathogens to their hosts, and can cause Lyme disease, Rocky-Mountain spotted fever, Colorado tick fever, babesiosis, ehrlichiosis, tularemia, and relapsing fever[1]. Twenty-four to 48 h of the ticks' attachment to the host are required before infection occurs, thus their early removal is useful in order to prevent diseases.

The application of dermoscopy to detect the tick in the lesion is easy, inexpensive, and effective for confirming the diagnosis[2,3].

### REFERENCES

1. Bratton, R.L. and Corey, G.R. (2005) Tick-borne disease. *Am. Fam. Physician* **71,** 2323–2332.
2. Oiso, N., Kawara, S., Yano, Y., and Kawada, A. (2010) Diagnostic effectiveness of dermoscopy for tick bite. *J. Eur. Acad. Dermatol. Venereol.* **24,** 231–232.
3. Zalaudek, I., Giacomel, J., Cabo, H., et al. (2008) Entodermoscopy: a new tool for diagnosing skin infections and infestations. *Dermatology* **216,** 14–23.

**This article should be cited as follows:**

Rallis, E. (2010) Dermoscopy of tick bite. *TheScientificWorldJOURNAL* **10**, 1705–1706. DOI 10.1100/tsw.2010.175.
